# Inflammation as a Prognostic Marker in Heart Failure

**DOI:** 10.7759/cureus.28605

**Published:** 2022-08-30

**Authors:** Priscila C Lima, Davi M Rios, Filipe P de Oliveira, Larissa R Passos, Ludmila B Ribeiro, Renato G Serpa, Osmar A Calil, Lucas C de Barros, Luiz Fernando M Barbosa, Roberto R Barbosa

**Affiliations:** 1 Cardiology, Hospital Santa Casa de Misericórdia de Vitória, Vitória, BRA; 2 Cardiology, Escola Superior de Ciências da Santa Casa de Misericórdia de Vitória, Vitória, BRA

**Keywords:** hospitalization, c-reactive protein, albumin, inflammation, prognosis, heart failure

## Abstract

Background: Heart failure (HF) is a chronic cardiac disease of great importance worldwide and responsible for one-fifth of hospitalizations for cardiovascular disease in Brazil. Pro-inflammatory mediators are involved in the pathophysiology of HF. However, the impact of inflammatory markers on the prognosis of the disease remains uncertain.

Objective: We aimed to evaluate inflammation as a prognostic marker in chronic HF.

Methods: In this prospective, single-center, observational cohort study conducted from June 2018 through December 2019, we included outpatients with HF from a specialized service of a teaching hospital. Patients with decompensated HF requiring hospitalization in the last 30 days were excluded. At the time of inclusion, serum C-reactive protein (CRP) and albumin were collected and the presence of inflammation was defined as CRP/albumin ≥1.2. Patients with CRP/albumin ratio <1.2 (group A) and CRP/albumin ratio ≥1.2 (group B) were compared. The primary outcome was all-cause mortality. The secondary outcomes were hospitalization for decompensated HF, number of hospitalizations, and number of days of hospitalization in the 12-month follow-up.

Results: We included 77 patients, 49 (63.3%) in group A and 28 (3.4%) in group B. Six patients in group A (12.2%) and 10 patients in group B (35.7%) required at least one hospitalization during follow-up (p=0.01). The rate of hospitalizations for decompensated HF for every 100 patients was 16.3 in group A vs 50.0 in group B (p=0.0001) and the average in-hospital length of stay was 12.2 vs 14.2 days per hospitalized patient (p=0.36) in groups A and B, respectively. The mortality rate was 6.1% in group A vs 7.1% in group B (p=0.86).

Conclusion: In HF outpatients with inflammation evidentiated by the CRP/albumin ratio ≥1.2, the risk of death was similar to patients without inflammation criteria. However, the presence of inflammation led to a three-fold higher risk of hospitalization for HF decompensation.

## Introduction

Heart failure (HF) is chronic heart disease with increasing prevalence and high socioeconomic impact [1]. In Brazil, HF is responsible for at least one-fifth of hospitalizations due to circulatory diseases [2]. The development of HF involves changes in several homeostatic systems. After tissue injury, the myocardium responds with remodeling and, in an adaptive process, there is an increase in the ventricular volume with a consequent increase in the final diastolic volume [3]. Neurohumoral mechanisms, such as activation of the adrenergic tonus, enhancement of the renin-angiotensin-aldosterone system, and release of the inflammatory cytokines initially are compensatory and try to maintain homeostasis, but persistent tissue injury leads to an imbalance. This imbalance leads to increased circulation levels of norepinephrine, angiotensin II, aldosterone, endothelin, vasopressin, and cytokines, which results in sodium and water retention and peripheral vasoconstriction with hemodynamic stresses on the ventricle, besides direct toxic effects on cardiac cells leading to myocardial fibrosis and for the final pathway: systolic ventricular dysfunction. This neurohormonal activation also changes the performance and phenotype of the myocytes and interstitium [1].

Recent studies have identified the importance of pro-inflammatory mediators in the development and progression of HF. The immune system reacts by removing apoptotic cells, inducing a systemic inflammatory response. The production and release of cytokines activate the complement system and recruit cells to the area of inflammation [4]. Therefore, HF can be considered a systemic disease with an important inflammatory component.

In chronic inflammatory processes, as in HF, there is an early increase in C-reactive protein (CRP), ferritin, and fibrinogen, positive acute phase proteins, and a delayed reduction of acute phase negative proteins, such as albumin, pre-albumin, and transferrin [1,5-6]. Based on this pathophysiology, a clinical prediction index for systemic inflammatory diseases has been described, the CRP/albumin ratio, which showed an association with higher mortality if ≥1.2 when compared to patients with a ratio of ˂1.2 [7].

There are no scientific data regarding this association in patients with HF until now. It is postulated that the presence of inflammation in HF is a marker of adverse events in clinical evolution. Thus, the present study aimed to assess inflammation, as measured by the CRP/albumin ratio, as a marker for prognostic stratification in patients with HF.

## Materials and methods

Study design

We conducted a prospective, single-center cohort study that evaluated the presence of CRP/albumin ratio ≥1.2 as a prognostic marker in patients with HF.

Patients

We included outpatients of both sexes with HF with reduced or mildly reduced ejection fraction (EF), thus <50% by the Simpson method on echocardiogram. Patients should have a regular follow-up at a specialized HF service of a teaching hospital. Patients with hospitalization for decompensated HF in the last 30 days before the time of inclusion, class IV of the New York Heart Association (NYHA), renal failure, liver disease, or with acute inflammatory disease were excluded.

Data collection

Patients were included from June 2018 through December 2019, and clinical data were collected based on the analysis of medical records. Information such as age, sex, comorbidities, etiology of HF, NYHA functional classification, and use of guideline-directed medical therapy (GDMT) in optimized doses was obtained at inclusion. Optimized treatment was considered as the use of GDMT (sacubitril-valsartan or angiotensin-converting enzyme inhibitors or angiotensin II receptor blockers, beta-blockers, and spironolactone) in target doses or maximum tolerated doses, as prescribed by the assistant physician. Serum CRP and albumin were collected at the time of inclusion in the study. The NYHA functional classification was assessed at the time of inclusion using information from medical records after clinical evaluation by the medical team. Patients were allocated into two groups according to the individual results of the CRP/albumin ratio. Group A included patients with a CRP/albumin ratio < 1.2 and group B included those with a CRP/albumin ratio ≥1.2.

Follow-up

Regular routine appointments at the HF ambulatory service occurred within a maximal interval of six months, according to the medical team criterion. Maintenance of the best GDMT was encouraged as a routine for medical staff and patients. Data regarding hospitalizations were collected directly from medical records. Patients who did not return to the outpatient routine visits were contacted by telephone to obtain information about clinical outcomes.

Outcomes

The primary clinical outcome analyzed was all-cause mortality assessed during a follow-up of 12 months. The secondary outcomes were hospitalization for decompensated HF, number of days of hospitalization, and length of hospital stay. We calculated the average length of hospitalization in each group by the ratio of days of hospitalization to the total number of hospitalized patients. Since cardiovascular death was thought to be frequently mistaken, we used only death from any cause as the primary outcome. Hospitalizations for HF were considered when HF decompensation was described as the admitting diagnosis.

Statistical analysis

Statistical Package for the Social Sciences (SPSS; IBM, Armonk, New York, USA) software version 23.0 was used for statistical analysis. Categorical variables were described as absolute and percentage frequency. Continuous variables were described as average and standard deviation, or as median and interquartile range (IQR) if not normally distributed. For comparative analyses, the Wilcoxon, the Mann-Whitney, and unpaired student t-tests were used, with p values less than 0.05 being considered statistically significant.

Ethical aspects

After clarifying the objectives and procedures to be performed, the research participants read and signed the Free and Informed Consent Form. The study was approved by the institution’s Research Ethics Committee, under number 087482. Ethical standards in clinical research were respected in accordance with the Declaration of Helsinki.

## Results

A total of 77 patients were enrolled in the study. Of these, 31 (40.3%) were female and 46 (59.7%) were male subjects, with a mean age of 59.9 ± 14.6 years, and a mean EF of 37.1% ± 11%. Regarding the evidence of inflammation, the mean values of CRP, albumin, and CRP/albumin ratio were, respectively, 9.3 mg/dl, 4.4 g/dl, and 2.38. The respective medians were 3.1 mg/dl (IQR=1.5-7.15), 4.5 g/dl (IQR=4.2-4.8), and 0.64 (IQR=0.31-1.71).

Of the patients analyzed, 49 (63.6%) had a CRP/albumin ratio <1.2 (Group A) and 28 (36.4%) had a ratio ≥ 1.2 (Group B). There were no significant differences between the two groups regarding sex, mean age, EF, presence of atrial fibrillation, and rate of use of beta-blockers, angiotensin-converting enzyme inhibitors (ACEI)/angiotensin II receptor blockers (ARB)/sacubitril-valsartan or spironolactone (Table 1). There were significant differences of CRP (3.1 vs 12.4 mg/dl, p=0.0001), serum albumin (4.5 vs 4.1 g/dl, p=0.048), and CRP/albumin ratio (0.69 vs 3.18) between groups A and B, respectively.

**Table 1:** Baseline clinical characteristics according to the absence (group A) or the presence (group B) of laboratory-demonstrated inflammation. ACEI: angiotensin-converting enzyme inhibitors; ARB: angiotensin II receptor blockers; GDMT: guideline-directed medical therapy

	Group A	Group B	P value
Age (years)	58.2 (± 14)	60.9 (± 13)	0.39
Female	21 (42.9%)	10 (35.7%)	0.53
Male	28 (57.1%)	18 (64.1%)	0.53
Ejection fraction	36.7% (± 11)	36.4% (± 12)	0.91
Use of beta-blockers	49 (100%)	28 (100%)	1
Use of beta-blockers at a target dose	40 (81.6%)	23 (82.1%)	1
Use of ACEI/ARB	39 (79.6%)	19 (67.8%)	0.25
Use of sacubitril-valsartan	8 (16.3%)	7 (25%)	0.35
Use of spironolactone	45 (91.8%)	26 (92.9%)	0.87
Use of GDMT	38 (77.5%)	21 (75%)	0.79
Presence of atrial fibrillation	14 (28.6%)	(28.6%)	1

There was no significant difference in mortality between groups during the 12-month follow-up: 6.1% in group A vs 7.1% in group B. Group A had a lower hospitalization rate and a lower total number of hospitalizations when compared with group B. Among patients who were hospitalized for decompensated HF, the mean hospital stay length was similar between groups A and B. Data related to outcomes are expressed in Table 2.

**Table 2:** Clinical endpoints at 12-month follow-up.

		Group A		Group B	P value
Mortality		3 (6.1%)		2 (7.1%)	0.86
Hospitalization rate		6 (12.2%)		10 (35.7%)	0.01
Total number of hospitalizations		8		14	0.0001
Length of hospitalization (days)		12.2		14.2	0.36

## Discussion

Several biomarkers have been described as risk predictors in HF, such as immune activation markers (e.g. Insulin-like growth factor 1 (IGF1), tumor necrosis factor (TNF), IL-6, CRP), natriuretic peptides (e.g., B-type natriuretic peptide (BNP) and N-terminal proBNP NT-proBNP), and high-sensitivity cardiac troponin [2]. In our study, we proposed the analysis of serum levels of CRP and albumin ratio as a risk predictor in HF outpatients. Inflammation, identified as an increase in the CRP/albumin ratio, was associated with a higher hospitalization rate due to HF decompensation. However, we found no significant association between the CRP/albumin ratio and mortality.

Albumin is a liver protein that in low values is associated with numerous deleterious biological processes present in the genesis of HF and that results in a worse prognosis [8]. A cohort of chronic HF patients followed for approximately 500 days demonstrated that a serum albumin value below 3.5 g/dl was a significant predictor of cardiovascular hospitalizations in patients with HF [9]. This information corroborates the results obtained in this present study, in which the group with lower serum albumin levels (group B) had higher rate of hospitalization. However, in our study, despite a significant difference between groups (4.5 vs 4.1 g/dl), the albumin value alone was not as clinically relevant as the CRP/albumin ratio. For the ratio, the role of albumin appears to have been smaller than that of CRP. It seems that further studies are needed to identify a cutoff value for albumin serum level as an independent prognostic marker.

For decades, it has been demonstrated that CRP is elevated in patients with HF, a heart disease that induces a systemic inflammatory response [10-12]. In addition, it is well established that the measurement of CRP, an exam that is easily obtained in healthcare services, although nonspecific, is able to predict adverse events in the long term [13]. In this perspective, a retrospective study demonstrated CRP as an independent predictor of hospitalizations in patients with HF [14]. As our study also suggests, CRP/albumin ratio is mostly changed by CRP levels, thus a prognostic impact of serum CRP values on patients with HF is expected. However, there are no consistent data to consider CRP as a modifiable risk factor. Although there is an association between CRP and hospitalization, the further reduction of CRP does not necessarily reduce the initial risk.

In our study, we observed a three-fold higher risk of hospitalization for decompensated HF in patients with this laboratory alteration. Thus, we ratify our initial hypothesis, in which patients with more signs of inflammation in the presence of HF have a greater number of decompensations requiring hospitalization. It is known that each hospitalization results in reduction of survival. Besides, the risk of a new hospitalization within six months after discharge is as high as 30 to 40% [15].

There was no significant difference in length of hospitalization between groups (12.2 days in group A and 14.2 days in group B), although these rates were higher than those found in studies from other centers. In the ADHERE study (Acute Decompensated Heart Failure National Registry), the mean length of hospital stay was 4.3 days [16]. Mortality in our study was similar between groups (6.1% in group A and 7.1% in group B), and both were also higher than that from ADHERE (4.0%), but lower than Brazilian data (12.6% in the Brazilian Registry of Heart Failure: Clinical Aspects, Care Quality and Hospitalization Outcomes (BREATHE)) [2,16].

It was not possible to establish an association between elevated inflammatory markers and mortality. However, studies have demonstrated that hypoalbuminemia and elevated CRP levels are important predictors of death in patients with HF in some scenarios. A study that enrolled 119 elderly patients admitted for decompensated HF showed that 42.3% of the patients who died had serum albumin levels ≤ 2.9 g/dl, while only 18.3% of the surviving patients had low serum albumin levels [17]. With regard to CRP, a retrospective analysis of the Valsartan Heart-Failure Trial demonstrated that patients with high CRP levels had a higher risk of death or the first morbid event [18]. The non-significant mortality difference in our study may be a result of the small sample size and follow-up time, and the wide use of optimized therapy, more than 75% of patients were on regular use of mortality-reducing drugs in combination. This may have softened the power of the study to assess this association by reducing the number of events.

Despite being an observational study and, therefore, not adjusted to confounding factors, the groups were similar regarding baseline clinical characteristics and pharmacological treatments offered. There was no significant difference in clinical variables such as age, sex, EF, presence of atrial fibrillation, use of beta-blockers, ACEI/ARB and spironolactone, and rate of adherence to these drugs, ensuring that all patients equally received the best treatment with disease-modifying medications.

As the sample was small, it was not possible to perform an adequate multivariate analysis. Furthermore, the power of the study cannot be measured. However as our study is observational, it aims to verify, in principle, an association of CRP/albumin ratio as a marker for prognostic stratification in HF, what has been demonstrated. To verify a causal relation, further studies are needed.

Some other limitations should also be noted. First, the serum concentration of CRP can be influenced by other factors, such as medications, smoking, alcohol consumption, chronic kidney disease, and other comorbidities. The data in this study were not adjusted for these factors. Second, our study was observational, and external validation cannot be assured. In addition, the cohort was based on a sample of patients followed up in an HF specialized service, making these results not applicable for HF patients followed up in primary care services.

## Conclusions

Outpatients with HF and high inflammation levels evidentiated by the CRP/albumin ratio ≥ 1.2 had higher rates of hospitalization for decompensation when compared to patients without inflammation. The presence of inflammation was not associated with increased mortality in HF in the 12-month follow-up in our study. However, the last association requires further investigation in larger similar studies.
